# Language eligibility criteria and enrolled racial and ethnic composition in US clinical trials: a cross-sectional study

**DOI:** 10.64898/2026.10.09.26365106

**Published:** 2026-10-12

**Authors:** Alexander M Tolas, Lina Du, Narayan Schutz, Paul Schmiedmayer, Jack W. O’Sullivan, Anders Johnson, Christopher T Longenecker, Ashish Sarraju, Fatima Rodriguez, Eugene Yang, Daniel Seung Kim

## Abstract

**BACKGROUND:**

Federal policy now requires sponsors to submit diversity action plans for clinical trials, yet the modifiable features of trial design associated with enrolled composition remain poorly characterized. English-language proficiency requirements are written into eligibility criteria and recorded as free text in a public registry, making them visible and, in principle, amenable to revision, but their relationship to enrolled composition has been examined only within single disease areas and in small samples of trials. We examined whether these requirements and documented language accommodation were associated with the racial and ethnic composition of enrolled participants.

**METHODS AND FINDINGS:**

We conducted a cross-sectional study of interventional trials registered on ClinicalTrials.gov with at least one US site and posted results, using a registry snapshot retrieved in October 2026. The primary cohort comprised the 44,487 trials with exclusively US sites, of which 21,924 (49.3%) reported baseline race and 17,434 (39.2%) reported baseline ethnicity with counts that reconciled with the posted denominator. Both exposures were classified from free-text eligibility criteria using regular expressions validated against independent physician review of 150 trials. For the English-language requirement classifier, sensitivity was 88.2% and specificity was 99.2%. The outcome was the trial-level proportion of enrolled participants in each category, analyzed using generalized linear models adjusted for intervention class, therapeutic area, sponsor, phase, enrollment, sites, start year, US Census region, and the population composition of the trial’s site states. An English-language proficiency requirement appeared in 5,736 trials (12.9%); its prevalence rose from 7.4% of trials starting in 2010 to 26.2% in 2023 (odds ratio [OR] per decade 2.91, P<0.001), whereas documented accommodation, an eligibility statement allowing participation in a language other than English (Spanish in 99.9% of flagged trials), remained rare (788 trials, 1.8%). The requirement was associated with lower enrolled proportions of Asian (OR=0.85, 95% CI 0.77-0.93, *P* =0.001) and Hispanic/Latinx participants (OR=0.85, 95% CI 0.79-0.91, *P* <0.001), with no evidence of an association for White participants (OR=0.99, 95% CI 0.95-1.04, *P* =0.75). Documented accommodation was associated with a higher proportion of Hispanic/Latinx participants (OR=3.23, 95% CI 2.82-3.71, *P* <0.001). Geographic adjustment did not change the statistical significance of the requirement associations and attenuated the accommodation association. The main limitations were the cross-sectional design, which precludes causal inference; the restriction of enrollment analyses to trials reporting baseline demographics, which were more recent than those without such reporting; and possible misclassification of automatically classified exposures.

**CONCLUSIONS:**

English-language proficiency requirements became more than three times as common between 2010 and 2023 and were associated with lower enrolled proportions of Asian and Hispanic/Latinx participants. Less common documented language accommodation was associated with a higher enrolled proportion of Hispanic/Latinx participants. Because these criteria are investigator-written and publicly recorded, they may be a tractable target for improving enrollment equity, although whether revising them changes who enrolls requires prospective evaluation.

